# Psychometric properties, Rasch analysis, and measurement invariance of the Turkish Brief Self-Control Scale in early adolescents: exploring the mediating role of responsibility in the self-control and patience association

**DOI:** 10.3389/fpsyg.2026.1829371

**Published:** 2026-05-05

**Authors:** Ali Gökalp, Servet Üztemur, Tuba Çengelci Köse

**Affiliations:** 1 Department of Educational Sciences, Gaziantep University, Gaziantep, Türkiye; 2 Department of Turkish and Social Sciences Education, Faculty of Education, Anadolu University, Eskişehir, Türkiye

**Keywords:** Brief Self-Control Scale, measurement invariance, patience, Rasch analysis, responsibility, Turkish early adolescents

## Abstract

**Introduction:**

This study aimed to rigorously evaluate the psychometric properties of the Brief Self-Control Scale (BSCS) for use with early adolescents aged 10–14 years within Turkish culture. Given developmental differences in cognitive and self-regulatory capacities during early adolescence, the study sought to determine whether the original scale structure is appropriate for this age group and cultural context, and to optimize the instrument for preliminary screening purposes.

**Methods:**

Data were collected from 351 secondary school students (mean age = 11.73; 50.4% girls). A dual analytical framework integrating ordinal confirmatory factor analysis (CFA) and Item Response Theory was employed. Rasch analysis using the Partial Credit Model was conducted to examine item-level functioning, threshold ordering, and developmental appropriateness. Items demonstrating poor psychometric performance or cognitive mismatch were diagnostically removed. Following item refinement, multigroup CFA was performed to test measurement invariance across gender at the configural, metric, scalar, strict, and structural levels. Concurrent and nomological validity were examined using the Responsibility and Patience Scales as external criteria. Predictive validity was further assessed through a latent mediation model within a structural equation modeling framework.

**Results:**

Rasch analysis identified four underperforming items, partly due to disordered threshold parameters indicating developmental incongruence for early adolescents, leading to their removal. The resulting 9-item scale demonstrated satisfactory model fit. Multigroup CFA confirmed full measurement invariance across gender. The refined BSCS-9 showed meaningful positive associations with responsibility and patience, supporting concurrent and nomological validity. In the latent mediation model, responsibility emerged as a significant mediator between self-control and related character traits, providing robust evidence for the predictive validity of the scale.

**Discussion:**

The findings establish the Turkish BSCS-9 as a parsimonious, developmentally appropriate, and psychometrically robust preliminary screening tool for assessing low-to-moderate levels of self-control in early adolescents. The integration of ordinal CFA with Rasch modeling offered a nuanced and methodologically consistent evaluation, highlighting the importance of aligning measurement instruments with developmental stage characteristics. Overall, this study supports the utility of the Turkish BSCS-9 in developmental and educational research contexts and demonstrates the value of combining modern psychometric approaches in scale adaptation and validation studies.

## Introduction

Various elements in an individual’s life shape their behavior. The legal, moral, traditional, cultural, and value systems of the society in which the individual lives influence their decision to engage in different behaviors. At this point, the concept of self-control is crucial for understanding why an individual exhibits or does not exhibit certain behaviors. Self-control refers to the ability to modify one’s reactions to align with standards such as morality, ideals, values, and social expectations, and to achieve long-term goals. Self-control allows a person to restrain or override a response, making a different response possible (Baumeister et al., 2007). Since self-control involves conscious efforts to prevent or alter a specific behavior before it occurs, it is also related to various concepts in developmental and social psychology that help regulate emotions, thoughts, and behaviors (Finkenauer et al., 2005). In today’s digital environment, this regulatory ability has become more essential than ever to reduce negative risk-taking behaviors, especially given the psychological effects of problematic social media use among adolescents (Liang et al., 2022; Simsir-Gokalp and Akyurek, 2024).

Research indicates that self-control involves both resisting problematic impulses and actively engaging in goal-oriented behaviors (Gökalp et al., 2026; Üztemur and Dinç, 2023). For example, resisting the urge to eat unhealthy foods is a form of self-control to maintain a healthy weight. Likewise, reaching that goal requires the proactive effort to exercise regularly (Blalock et al., 2022; Schrader and Tangney, 2024). Especially in education, high levels of self-control are essential for students to stay focused academically and avoid risky behaviors, ultimately fostering a more effective learning environment. In tasks and questionnaire items designed to measure self-control as a factor influencing individuals’ decisions and behaviors across various domains, a response is considered better if it aligns with the individual’s long-term goals and standards. Self-controlled individuals tend to choose the better response, while impulsive individuals often opt for the immediately satisfying or automatic response (Duckworth and Kern, 2011). The literature shows that the “Brief Self-Control Scale” developed by Tangney et al. (2004) is widely used for measuring self-control and has been adapted for different age groups and cultures (Bertrams and Dickäuser, 2009; Brevers et al., 2017; Chen et al., 2022; Chiesi et al., 2020; Rodríguez-Menchón et al., 2022; Torres-Marín et al., 2024; Unger et al., 2016; Zhang et al., 2021). The BSCS, adapted for Turkish adults, includes two sub-dimensions (self-discipline and impulsivity) with nine items (Nebioglu et al., 2012). Rodríguez-Menchón et al. (2022) studied the factorial structure of the BSCS among Spanish adolescents aged 13–18. Another study (Chen et al., 2022) translated the BSCS into Chinese for adolescents aged 11–19, resulting in a unidimensional structure with 12 items. To our knowledge, no research has been conducted on the Turkish version of the BSCS for secondary students aged 10–14. This study therefore aims to adapt the BSCS to Turkish culture and to fill this gap in the literature.

The secondary school years are generally seen as the beginning of adolescence. Adolescence, which marks the transition from childhood to adulthood (Casey, 2015), is a key stage in developing personality. During this time, identity formation continues as a process of building a consistent sense of self (Damon and Gregory, 1997). Adolescents develop self-awareness and begin to form a perspective that guides their actions. In this period, their awareness of self-control is crucial for deciding whether to exhibit certain behaviors. The literature review shows that various studies have explored the relationship between adolescent self-control and different variables (Finkenauer et al., 2005; Kuhnle et al., 2012; Svensson et al., 2010). Additionally, developing self-control during these formative years lays a psychological foundation that helps adolescents handle the growing academic and social challenges of secondary education.

The current study also examined the relationship between secondary school students’ self-control levels in early adolescence and their levels of responsibility and patience. Responsibility is a complex and challenging concept to define. Schlenker et al. (1994) proposed the triangle model of responsibility. This model highlights that “responsibility is the adhesive that connects an actor to an event and to relevant prescriptions that should govern conduct; thus, responsibility provides a basis for judgment and sanctioning” (Schlenker et al., 1994, p. 635). The development of responsibility as a value can be viewed within the context of moral development. According to Piaget, moral development involves two main stages: the externalized and autonomous periods. In the externalized stage, moral realism predominates. In the autonomous stage, individuals accept that rules can change depending on the situation. At the autonomous stage, individuals are expected to have learned and internalized rules (Akçinar et al., 2018). For an individual’s value of responsibility to become internalized, the person must think abstractly in cognitive terms and progress toward the autonomous moral stage. At the secondary school level, children can internalize the value of responsibility through both cognitive and moral development. However, individual differences and influences from family and social environments can cause variations in how people internalize and enact the value of responsibility. Since individuals with high self-control have a greater capacity to regulate their behavior, it is important to explore the relationship between self-control and the value of responsibility. Recent theoretical approaches emphasize that self-control functions as the internal mechanism that enables adolescents to act on their internalized sense of responsibility, reducing psychological distress and promoting ethical decision-making (Koç et al., 2023).

Another concept linked to self-control is patience. Patience is the ability or capacity to wait calmly without becoming angry or acting disruptively (Vigani, 2017). Besides waiting calmly, patience also involves using this trait to manage stressors like life challenges and daily interpersonal issues. Additionally, patience can affect how a person responds to frustration (Shubert et al., 2022). Therefore, patience is not just a passive trait but an active, self-regulatory virtue that helps adolescents navigate academic and social hurdles effectively. In this context, exploring the connections among self-control, patience, and responsibility is expected to yield valuable insights for the literature. This study aims to adapt the self-control scale to Turkish culture, using a sample of secondary school students (ages 10–14), to better understand adolescent behaviors, support character development, and assess adolescents’ levels of self-control, responsibility, and patience. Furthermore, to validate the scale’s nomological and predictive validity within a complex network of character traits, this study incorporated these theoretically related variables (responsibility and patience) into a latent mediation model. Based on the outlined theoretical frameworks, the following specific hypotheses were developed for the mediation model:

Hypothesis 1 (H1): Self-control will positively predict patience among early adolescents.

Hypothesis 2 (H2): Responsibility will significantly mediate the relationship between self-control and patience.

Theoretically, this mediation is expected because self-control provides the basic mental ability to manage impulses, while responsibility functions as an internal moral system that guides this ability toward consistent, value-driven patience.

## Method

### Participants, recruitment procedures, and ethical considerations

The study involved 351 Turkish students (50.4% girls) randomly selected from different secondary schools during the 2024–2025 academic year. Data were collected after obtaining approval from the research ethics committee and the research application committee. The study included only students with parental consent. Researchers gathered the data face-to-face using paper and pencil. Participants were reminded to answer each question carefully. Besides the characteristics listed in Table 1, participants were not asked any questions that could identify them (e.g., name, school name). Participation was voluntary, and no compensation was provided. Detailed demographic information, including age distribution, parental cohabitation status, grade levels, and parental education backgrounds, is shown in Table 1.

**Table 1:** Participants’ characteristics (*N* = 351).

Characteristics	Mean or *n*	SD or %
Age	11.73	1.45
Gender		
Girl	177	50.4%
Boy	174	49.6%
Cohabitation status of parents		
Yes	312	88.9%
No	39	11.1%
Grade level		
5th	99	28.2%
6th	82	23.4%
7th	92	26.2%
8th	78	22.2%
Mother graduation		
Primary	89	25.4%
Secondary	66	18.8%
High school	81	23.1%
College	115	32.8%
Father graduation		
Primary	46	13.1%
Secondary	64	18.2%
High school	100	28.5%
College	141	40.2%

SD, standard deviation. The participants ranged in age from 10 to 14 years (*M*_age_ = 11.73, SD = 1.45).

### Measure adaptation

The Brief Self-Control Scale (Tangney et al., 2004), adapted for Turkish culture, contains nine items (see Appendix A). The steps outlined by Hambleton (2005) for the linguistic equivalence process were followed to determine whether the items in the original language of the BSCS were linguistically equivalent to those in the translation. First, a translation team with strong proficiency in both English and Turkish was assembled. For translating the scale into Turkish, the English version was sent to three independent language experts who are fluent in both languages and knowledgeable in their respective fields. Then, the Turkish version of the scale was finalized by selecting the translation from a different language expert that most accurately reflected the meaning of each item. The items are rated on a five-point scale (1 = Not at all suitable for me, 5 = Very suitable for me). The nine items were reverse-coded so that higher scores indicated greater self-control (see Appendix A). Then, the scale’s construct, criterion, and reliability were analyzed using data from 351 secondary students, whose characteristics are presented in Table 1.

### Other measures

To evaluate the psychometric strength of the Turkish BSCS-9, responsibility and patience were used in two ways. First, they acted as external criteria to establish concurrent validity through bivariate correlations, measuring how well the scale aligns with theoretically related constructs in early adolescence. Second, these variables were incorporated into a latent mediation model to test the scale’s nomological and predictive validity. This method allowed us to assess not only the instrument’s accuracy in reflecting the self-control construct but also its practical usefulness within a complex system of character traits.

The seven-item Responsibility Scale (Gökalp, 2021) was used to assess responsibility. The items of the unidimensional Responsibility Scale are evaluated on a four-point scale (1 = does not describe me at all; 4 = describes me completely). Higher scores indicate greater levels of responsibility (Sample item: “I always do what I promise to do”). In the current study, the internal consistencies of the Responsibility Scale were acceptable (Cronbach’s *α* = 0.702; McDonald’s *ω* = 0.706). In addition, the fit indices obtained from confirmatory factor analysis (CFA) were good: RMSEA = 0.034, SRMR = 0.029, CFI = 0.980, TLI = 0.970, IFI = 0.980.

The six-item Patience Scale (Gökalp, 2022) assessed patience. The unidimensional “Patience Scale” items are evaluated on a four-point scale (1 = does not describe me at all; 4 = describes me completely). Higher scores indicate greater patience (Sample item: “When my parents say they will buy something I like later, I wait and do not insist.”). In the present study, the internal consistencies of the Patience Scale were acceptable (Cronbach’s *α* = 0.724; McDonald’s *ω* = 0.732). In addition, the fit indices obtained from CFA were sufficient: RMSEA = 0.063, SRMR = 0.047, CFI = 0.948, TLI = 0.913, IFI = 0.949.

### Data analysis

The data collected from the students were carefully entered into the Statistical Package for the Social Sciences (SPSS) for analysis. CFA was conducted to verify whether the original unidimensional structure of the scale was retained in the Turkish version. To ensure that the scale measures the same underlying construct equivalently across gender groups, a multi-group confirmatory factor analysis (MG-CFA) was conducted to test for measurement invariance. Addressing methodological necessities for accurate latent mean comparisons, this process involved a series of increasingly restrictive models: configural invariance (testing the same factor structure), metric invariance (testing equal factor loadings), scalar invariance (testing equal item intercepts), strict invariance (testing equal residual errors), and structural invariance (testing equal latent variances and covariances). According to Chen (2007), a change in the Comparative Fit Index (ΔCFI) of less than 0.01 and a change in the Root Mean Square Error of Approximation (ΔRMSEA) of less than 0.015 were used as criteria to support invariance at each sequential step.

CFA, MG-CFA, and internal consistency analyses were conducted using Jeffrey’s Amazing Statistics Program (JASP), while other analyses utilized SPSS. Since the 5-point Likert-type items are ordinal variables, the factor analyses were strictly carried out using the robust Weighted Least Squares Mean and Variance Adjusted (WLSMV) estimator. This method does not assume multivariate normality and avoids biased factor loadings or inflated fit indices often linked to Maximum Likelihood (ML) estimation. To properly address this ordinal nature, the polychoric correlation matrix was used as the suitable input for the robust WLSMV estimation (see Appendix C). The following fit indices calculated from the CFA were used to determine acceptable model fit: comparative fit index (CFI) > 0.90, incremental fit index (IFI) > 0.90, Tucker-Lewis index (TLI) > 0.90, root mean square error of approximation (RMSEA) < 0.08, and standardized root mean square residual (SRMR) < 0.08 (Kline, 2015). To provide a rigorous psychometric justification for the model evaluation, especially given the use of the robust WLSMV estimator and the polychoric correlation matrix for ordinal data, the econometric structures of these goodness-of-fit indices, which measure the exact discrepancy between the observed sample parameters and the model-implied parameters, are outlined below.

$$\mathrm{CFI}=1-\frac{\max(\chi_{M}^{2}-{\mathrm{df}}_{M},0)}{\max(\chi_{M}^{2}-{\mathrm{df}}_{M},\chi_{B}^{2}-{\mathrm{df}}_{B},0)}$$

$$\mathrm{TLI}=\frac{\left(\frac{\chi_{B}^{2}}{{\mathrm{df}}_{B}})-(\frac{\chi_{M}^{2}}{{\mathrm{df}}_{M}}\right)}{(\frac{\chi_{B}^{2}}{{\mathrm{df}}_{B}})-1}$$

$$\mathrm{IFI}=\frac{\chi_{B}^{2}-\chi_{M}^{2}}{\chi_{B}^{2}-{\mathrm{df}}_{M}}$$

$$\text{SRMR}=\sqrt{\left(\frac{\sum {\left(r_{\mathrm{ij}}-{\hat{r}}_{\mathrm{ij}}\right)}^{2}}{\frac{p(p+1)}{2}}\right)}$$

$$\text{RMSEA}=\sqrt{\left(\frac{\max(\chi_{M}^{2}-{\mathrm{df}}_{M},0)}{\left({\mathrm{df}}_{M}\;(N-1)\right)}\right)}$$

Where *χ*^2^*_M_* and df*_M_* denote the robust WLSMV chi-square statistic and adjusted degrees of freedom for the hypothesized model; *χ*^2^*_B_* and df*_B_* represent those for the baseline/null model; *N* is the sample size; *r_ij_* and *r̂_ij_* reflect the observed polychoric correlations and model-implied components, and *p* is the number of observed items. The factor loading values obtained from the CFA should be >0.30 (Harrington, 2009). In addition, a good scale is expected to have a corrected item-total correlation value of 0.30 or more (Cristobal et al., 2007). Furthermore, to thoroughly evaluate the psychometric properties at the item level and to provide a diagnostic assessment of items with inadequate factor loadings, a Rasch analysis using the Polytomous Partial Credit Model (PCM) was conducted. The Rasch parameters were estimated via Marginal Maximum Likelihood Estimation (MMLE) using the snowIRT module in Jamovi. By employing the robust WLSMV estimator for the CFA alongside the PCM for the Rasch analysis, both models appropriately treated the 5-point Likert items as ordinal variables. This dual framework effectively resolved the methodological incompatibility often encountered when mixing traditional continuous CFA with non-linear Item Response Theory (IRT) models.

Before analyzing the Rasch parameters, the essential assumptions of local independence and unidimensionality were thoroughly tested using Yen’s Q3 statistic and the Mean Absolute Deviation of aQ3 (MADaQ3). Furthermore, to evaluate the scale’s structural unidimensionality within the Rasch framework, a Principal Component Analysis (PCA) of Rasch residuals was conducted to examine the eigenvalue of the first contrast (*λ*_1_). To ensure high computational precision for this structural assumption, the PCA of residuals was performed using the TAM (Test Analysis Modules) package (Robitzsch et al., 2023) in the R programming environment (R Core Team, 2023) via Google Colaboratory. Subsequently, Person and Item Reliability, as well as Item Separation indices, were calculated to assess the scale’s ability to discriminate along the latent continuum. The performance of individual items was checked using Infit and Outfit mean-square statistics. Lastly, to rigorously evaluate whether the scale items function equivalently across genders within the IRT framework, a true Differential Item Functioning (DIF) analysis was conducted. Using the difNLR package, likelihood ratio chi-square statistics specifically designed for ordinal data were estimated, and the resulting *p*-values were adjusted for multiple comparisons (*padj*). Following the item refinement process, the final scale was examined for internal consistency using Cronbach’s *α* and McDonald’s *ω*. A value >0.70 in McDonald’s *ω* or Cronbach’s *α* indicates good internal consistency (George and Mallery, 2016).

The scale was analyzed for concurrent validity with relevant external criteria (i.e., the Responsibility and Patience scales) using Pearson correlations. Additionally, scores were assessed to determine if they varied significantly across sociodemographic variables (gender, parent cohabitation status, grade level, and parents’ education level) using independent t-tests and one-way ANOVA with Bonferroni correction. Finally, to address methodological limitations and potential measurement errors, the mediating role of responsibility between self-control and patience was tested using a comprehensive Structural Equation Modeling (SEM) framework rather than a simple observed-variable regression approach. Specifically, self-control, responsibility, and patience were modeled as latent variables with their respective measurement errors. To ensure consistency with the initial CFA, this latent mediation SEM model was also estimated using the robust WLSMV estimator. The significance of the indirect effects within the SEM was evaluated, and an indirect effect was considered significant if the 95% confidence interval’s upper and lower limits did not include zero (Hayes, 2022).

## Results

Table 2 provides a detailed item-level evaluation of the scale by combining metrics from both CFA and the Rasch model. Specifically, the table shows the strong WLSMV factor loadings, standard reliability coefficients, and Rasch Infit/Outfit statistics to confirm the structural validity and proper functioning of the ordinal items.

**Table 2:** Factor loadings, Rasch fit statistics, and frequentist individual item reliability for the BSCS-9.

Items	WLSMV estimate	Infit	Outfit	*α* if the item dropped	Item-total correlation
2. I have a hard time breaking bad habits	0.479	1.033	1.034	0.717	0.406
3. I am lazy	0.468	1.112	1.135	0.720	0.392
4. I say inappropriate things	0.502	0.972	0.997	0.713	0.432
5. I do certain things that are bad for me if they are fun	0.656	0.972	0.900	0.696	0.539
6. I wish I had more self-discipline	0.375	1.235	1.260	0.733	0.320
7. Pleasure and fun sometimes keep me from getting work done	0.381	1.003	1.072	0.730	0.324
8. I have trouble concentrating	0.509	1.038	1.048	0.710	0.450
10. Sometimes, I cannot stop myself from doing something, even if I know it is wrong	0.543	0.961	0.974	0.710	0.446
11. I often act without thinking through all the alternatives	0.520	1.109	1.129	0.712	0.437

WLSMV, Weighted Least Squares Mean and Variance Adjusted; Infit, information-weighted mean square statistic; Outfit, outlier-sensitive mean square statistic. Factor loadings represent completely standardized estimates (std. all) for the final 9-item model. All estimates are statistically significant at the *p* < 0.001 level. The Rasch model yielded a Person Reliability of 0.889. Additionally, the Item Reliability was 0.97, and the Item Separation Index was 5.78, indicating robust separation of items along the trait continuum.

The factor loadings of the BSCS items obtained from the robust WLSMV estimation ranged from 0.465 to 0.868, although four items (item 1, item 9, item 12, and item 13) had factor loadings below 0.30 (see Appendix A). Specifically, the WLSMV estimation indicated that the factor loading for Item 13 (0.123) was not statistically significant (*p* = 0.054). Additionally, beginning with the item with the lowest corrected item-total correlation coefficient (item 13 = 0.145), items were sequentially removed (item 9 = 0.183; item 12 = 0.190; item 1 = 0.177). As a result of these steps, the item analysis revealed corrected item-total correlations ranging from 0.320 to 0.539, and the final WLSMV factor loadings ranged from 0.375 to 0.656 for the BSCS-9 (Table 2).

To further evaluate the psychometric robustness of the scale and provide a deeper methodological justification for the exclusion of four items (Items 1, 9, 12, and 13) which had previously demonstrated weak factor loadings (<0.30) in the robust WLSMV estimation, a Rasch analysis utilizing the Partial Credit Model (PCM) was conducted. Prior to examining item parameters, the assumption of local item independence was tested using Yen’s Q3 statistic. The Mean Absolute Deviation of aQ3 (MADaQ3) was low (0.0589, *p* < 0.001), and the Q3 correlation matrix revealed no substantial residual dependencies among the items, confirming the local independence assumption. Furthermore, to rigorously evaluate the scale’s structural unidimensionality, a PCA of Rasch residuals was conducted. The results indicated that the eigenvalue of the first contrast (*λ*_1_) was 1.426 (accounting for 15.85% of the variance). Because this value is well below the critical threshold of 2.0, it demonstrates that the implied dimension in the data has the strength of less than two items, confirming that no significant secondary dimensions distorted the BSCS-9 (Brentani and Golia, 2007; see Appendix D for full PCA results). This structural unidimensionality is additionally supported by the excellent fit indices obtained from the robust WLSMV CFA (as detailed in Table 3), thereby demonstrating the complementary strength of our dual-framework approach.

**Table 3:** Scale properties of the BSCS-9.

Confirmatory factor analysis (WLSMV)						Reliability	
*χ*^2^ (df)	CFI	TLI	IFI	RMSEA	SRMR	Cronbach’s *α*	McDonald’s *ω*
29.00 (27)	0.997	0.996	0.997	0.015	0.045	0.739	0.743

*χ*^2^, Chi-square; df, degrees of freedom; *p* = 0.361 for the exact fit test; CFI, comparative fit index; TLI, Tucker-Lewis index; IFI, incremental fit index; RMSEA, root mean square error of approximation; SRMR, standardized root mean square residual. All fit indices were estimated using the robust WLSMV estimator.

The initial analysis of the 13-item version showed a Person Reliability of 0.905. However, an examination of the Wright Map (Person-Item Map) revealed that Items 1, 9, 12, and 13 were located at significantly lower difficulty levels compared to the sample’s latent trait distribution, offering limited information. To clearly justify the exclusion of these underperforming items, the Category Probability Curves (CPC) for the removed items (Items 1, 9, 12, and 13) are provided in Appendix B. As shown in these curves, these items consistently displayed disordered threshold (*τ*) parameters, where multiple response categories did not emerge as the most probable choice at any point along the latent trait. After excluding these four underperforming items, a final Rasch analysis was conducted on the remaining 9 items (BSCS-9). The final model produced a Person Reliability of 0.889, demonstrating the scale’s sufficient ability to distinguish individuals along the latent logit continuum. Additionally, an excellent Item Reliability of 0.97 and an Item Separation Index of 5.78 were obtained, indicating that the sample was highly capable of accurately establishing the difficulty hierarchy of the items.

Regarding item targeting, the Wright Map showed that the item difficulties of the BSCS-9 ranged from −2.14 to −1.36 logits. As indicated by the targeting distribution, although all items are located in the lower part of the trait relative to the overall sample’s ability, this essentially means that the scale provides optimal discrimination mainly for early adolescents with low to moderate self-control levels, effectively identifying those at the highest risk for behavioral issues. Finally, unlike the severely disordered initial model, the tau parameters for the 9-item version operated within a much more stable range (6.10–8.15) and demonstrated significantly better category transitions for this age group. Together, these findings psychometrically support the BSCS-9 as a simple and developmentally suitable tool for measuring self-control in Turkish early adolescents.

Table 3 shows the strong WLSMV-based CFA results for the BSCS-9. The unidimensional structure of the BSCS-9 was clearly confirmed by excellent model fit indices (*χ*^2^/df ratio close to 1.0, CFI and TLI above 0.95, RMSEA and SRMR below 0.05), indicating that the 9-item model fits the ordinal data well. Additionally, the internal consistency of the BSCS-9 was assessed, producing acceptable reliability coefficients for early adolescents.

Table 4 shows the solid WLSMV-based multi-group CFA results for the measurement invariance of the BSCS-9 across gender groups. To ensure accurate comparisons of latent means, the models were tested in sequence, confirming configural, metric, scalar, strict, and structural invariances through acceptable change values based on the fit indices (ΔCFI < 0.01 and ΔRMSEA ≤ 0.015). Most importantly, establishing strict invariance (equal residual errors) means the scale measures the underlying construct consistently for both girls and boys, so any gender differences in self-control scores reflect true latent mean differences rather than measurement bias. Additionally, confirming structural invariance (equal latent variances and covariances) shows that the variation of the self-control construct is exactly the same across genders.

**Table 4:** Measurement invariance of the BSCS-9 across gender using the robust WLSMV estimator.

Model	*χ* ^2^	*df*	CFI	RMSEA	ΔCFI	ΔRMSEA	Decision
Configural Invariance	42.30	54	1.000	0.000	–	–	Supported
Metric Invariance	51.89	62	1.000	0.000	0.000	0.000	Supported
Scalar Invariance	68.05	70	1.000	0.000	0.000	0.000	Supported
Strict Invariance	74.63	79	1.000	0.000	0.000	0.000	Supported
Structural Invariance	84.34	81	0.995	0.015	−0.005	0.015	Supported

*χ*^2^, chi-square; df, degrees of freedom; CFI, comparative fit index; RMSEA, root mean square error of approximation. Changes in fit indices (ΔCFI < 0.01 and ΔRMSEA ≤ 0.015) are used as the criteria for establishing measurement invariance at each sequential step. All models were estimated using the robust WLSMV approach.

To further scrutinize the measurement invariance at the item level, a Rasch-based DIF analysis was conducted. The likelihood ratio test results, using *p*-values adjusted for multiple comparisons (*padj*), revealed that eight out of nine items showed no significant gender-based bias (*padj*​ > 0.05). Specifically, the values for these items ranged from *padj*​ = 0.131 to *padj*​ = 0.935. Although Item 5 displayed a statistically significant DIF (*padj*​ = 0.040), the effect was marginal and close to the threshold, suggesting that the overall scale maintains its functional equivalence across genders. These findings reinforce the scalar invariance established through MG-CFA (see Appendix E).

Analysis of Table 5 shows that BSCS is positively correlated with the Responsibility and Patience Scales. There is also a positive correlation between the Responsibility and Patience scales.

**Table 5:** Concurrent validity of the BSCS-9.

Characteristics	Pearson correlation with an external criterion measure^a^		
	BSCS-9	Responsibility scale	Patience scale
BSCS-9	—	0.194	0.258
Responsibility scale		—	0.582
Patience scale			—
Mean (SD)	3.20 (0.79)	3.09 (0.56)	2.99 (0.62)
Skewness	−0.24	−1.32	−0.93
Kurtosis	−0.18	1.67	1.27

a All correlations are significant at *p* < 0.001 level; BSCS-9, Brief Self-Control Scale; SD, standard deviation.

Table 6 shows no statistically significant differentiation in the BSCS-9 variable in gender groups, cohabitation status of parents, and parents’ graduation level groups. However, there is a statistically significant difference in grade-level groups. This difference between 6th and 8th grade favors 6th grade (6th mean = 3.36 > 8th mean = 2.97).

**Table 6:** Comparing the BSCS-9 between gender, cohabitation status of parents, grade level, and mother–father graduation level.

Mean (SD) in gender		*t* (*p*)	Mean (SD) in grade-level				*F* (*p*)
Girl	Boy		5th	6th	7th	8th	
3.13 (0.81)	3.27 (0.77)	1.71 (0.09)	3.25 (0.74)	3.36 (0.78)	3.23 (0.83)	2.97 (0.79)	3.73 (0.01)^a^
Mean (SD) in the cohabitation status of parents			Mean (SD) in the mother graduation level				
Yes	No		Primary	Secondary	High school	College	
3.22 (0.80)	3.09 (0.79)	0.93 (0.36)	3.19 (0.79)	3.19 (0.77)	3.15 (0.80)	3.25 (0.82)	0.28 (0.85)
			Mean (SD) in father’s graduation level				
			Primary	Secondary	High school	College	
			3.13 (0.70)	3.12 (0.79)	3.20 (0.82)	3.27 (0.81)	0.66 (0.58)

SD, standard deviation.

a Indicates a significant difference between 6th and 8th using Bonferroni adjustment.

Furthermore, these cross-group comparisons are based on observed mean scores, theoretically justified by the established measurement invariance.

**Figure 1:**
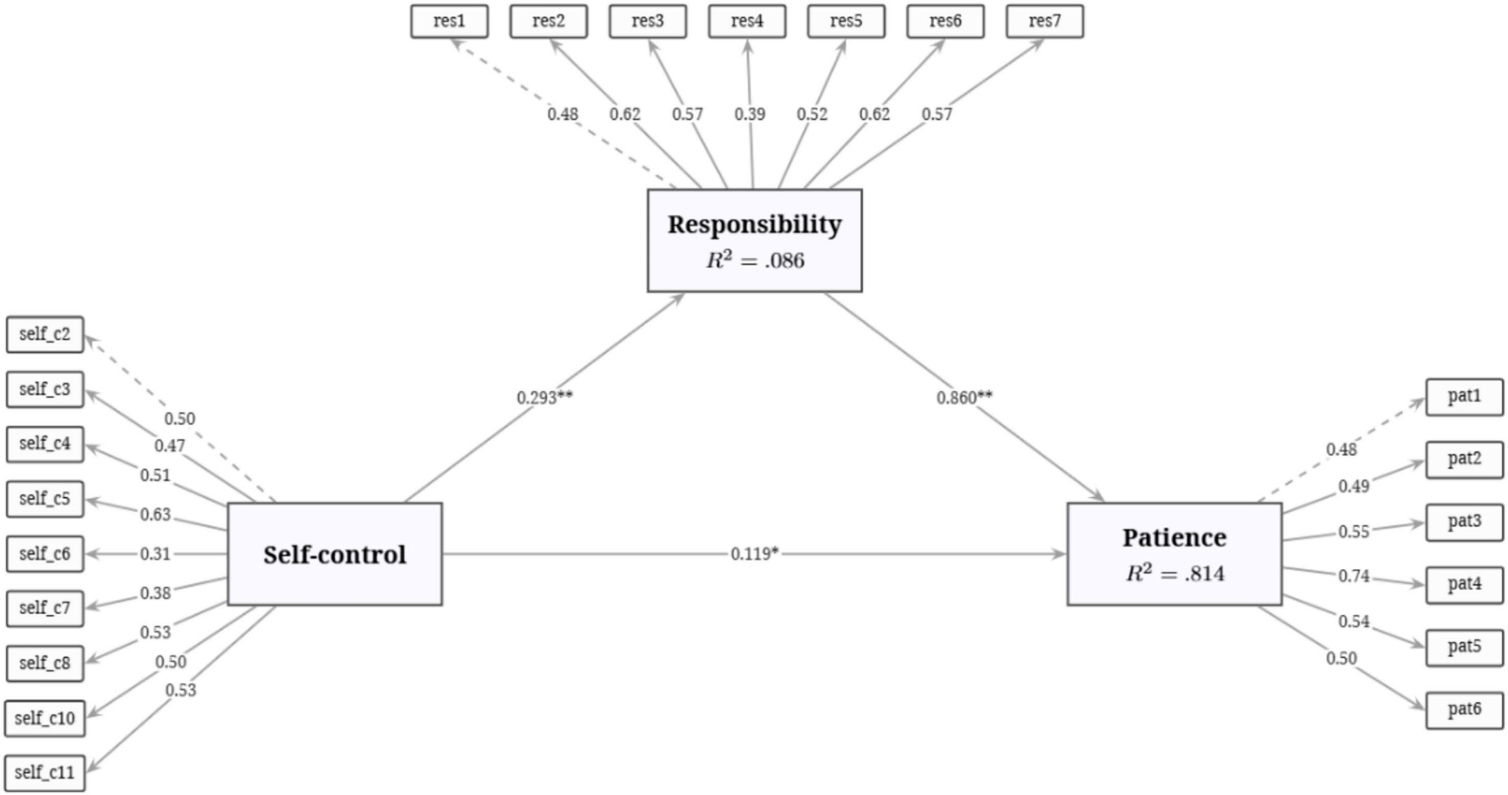
Path coefficients for the mediation model. *N* = 351. Standardized parameter estimates are presented. The robust WLSMV estimator was used. Model fit indices: *χ*^2^ (206) = 360.00, CFI = 0.971, TLI = 0.968, RMSEA = 0.046, SRMR = 0.057. ^*^p < 0.05. ^**^p < 0.001.

Figure 1 presents the standardized coefficients for the mediation model, which demonstrated a good fit to the data (*χ*^2^ (206) = 360.00, CFI = 0.971, TLI = 0.968, RMSEA = 0.046, SRMR = 0.057). Self-control positively predicted responsibility (*β* = 0.293, *z* = 3.47, *p* < 0.001), and 8.6% of the variance in responsibility was explained by self-control (*R*^2^ = 0.086), indicating a statistically significant but modest effect. Responsibility positively predicted patience (*β* = 0.860, *z* = 7.20, *p* < 0.001).

As detailed in Table 7, the total effect of self-control on patience was statistically significant (*β* = 0.372, *p* < 0.001). Therefore, H1 was supported. When responsibility was included as a mediator, the direct effect of self-control on patience was substantially reduced but remained significant (*β* = 0.119, *p* = 0.033). The significance of the indirect effect was confirmed, as the 95% confidence interval limits did not include zero [*β* = 0.252, 95% CI (0.136, 0.369)], indicating that responsibility significantly mediated the relationship between self-control and patience. Thus, H2 was supported. Strikingly, by explicitly modeling measurement errors through a full latent SEM framework, self-control and responsibility collectively explained 81.4% of the variance in patience (*R*^2^ = 0.814), providing robust empirical support for the theoretical model.

**Table 7:** Standardized indirect, direct, and total effects for the latent mediation model.

Effects	Path ways	*β*	*z*	*p*	95% CI [LL]	95% CI [UL]
Indirect effects	SLC → RSP → PTN	0.252	3.501	<0.001	0.136	0.369
Direct effects	SLC → PTN	0.119	2.130	0.033	0.019	0.220
Total effects	SLC → PTN	0.372	3.953	<0.001	0.236	0.507

CI, confidence Interval for the standardized beta coefficients; LL, lower LEVEL; UL, upper level; SLC, self-control; RSP, responsibility; PTN, patience. All parameters were estimated using the robust WLSMV approach.

## Discussion

The BSCS-13 (Tangney et al., 2004) has undergone validity and reliability testing across cultures and age groups. While representing a preliminary step in the validation process, this study proposes a nine-item Turkish adolescent version consisting of a single-factor structure. The proposed one-factor structure is consistent with results from other research (Bertrams and Dickäuser, 2009; Brevers et al., 2017; Chen et al., 2022). Crucially, the structural unidimensionality of this finalized version was stringently corroborated by both the excellent fit indices of the ordinal CFA and the robust evidence from the PCA of Rasch residuals (eigenvalue, *λ*_1_ < 2.0). From a methodological perspective, this study addresses the historical “metric gap” between Rasch modeling and CFA to clarify these structural debates. By using a strong ordinal CFA (WLSMV) along with the Rasch Partial Credit Model, it was ensured that both frameworks viewed the Likert data as inherently non-linear and ordinal. This combination resolves the conceptual inconsistency often seen in studies that blend logistic IRT scales with linear ML-based CFA. The findings demonstrate that when both models are grounded in ordinal assumptions, they provide a more precise diagnostic tool for assessing complex constructs like self-control in early adolescents. However, it is important to explicitly state that this integration is complementary, not formally unified. While our framework bridges the conceptual gap by treating Likert data as inherently non-linear in both analyses, there remains no real formal integration between IRT and SEM paradigms. However, the literature shows mixed results regarding the factor structure of the BSCS. For example, two dimensions were identified in the Turkish adult version (Nebioglu et al., 2012) and the Spanish adolescent version (Rodríguez-Menchón et al., 2022).

Apart from the factor structure, a key methodological contribution of this study is establishing full measurement invariance across gender groups. The results for configural, metric, scalar, strict, and structural invariance show that the nine-item Turkish BSCS-9 measures the core concept of self-control consistently for both girl and boy adolescents. This provides a theoretical justification for our observed mean comparisons (e.g., independent *t*-tests), ensuring that any observed gender differences in self-control scores reflect true construct differences rather than measurement bias (Vandenberg and Lance, 2000). Furthermore, confirming the structural invariance shows that the dispersion of the self-control construct is exactly the same across genders, offering the strongest evidence for cross-group comparisons. The inclusion of Rasch-based DIF analysis further bolsters the measurement invariance findings at the item level. The fact that eight out of nine items exhibited complete item neutrality confirms the suitability of the BSCS-9 for gender-based comparisons. The marginal *padj* value for Item 5 is considered negligible and does not compromise the overall scale integrity. This dual approach, integrating both MG-CFA and Rasch-based DIF, provides robust evidence that the scale maintains functional equivalence not only structurally but also at the item-parameter level, addressing the methodological nuances highlighted in recent psychometric literature.

The psychometric necessity of excluding four items highlighted by the Rasch analysis also warrants interpretation from a developmental cognitive perspective. Specifically, the disordered threshold parameters observed in Item 13 (“People around me say I have an iron will”) indicate a cognitive mismatch between the original item phrasing and the developmental stage of early adolescents. During the 10–14 age period, students are navigating the transition from concrete to formal operational thinking. Evaluating oneself through metaphorical expressions such as an “iron will” requires advanced abstract reasoning and the capacity to accurately gauge complex external social evaluations. This cognitive demand likely caused the inconsistent category transitions on the five-point scale. Thus, the Rasch results not only support the statistical refinement of the scale but also empirically demonstrate that some abstract items designed for adults do not function optimally for early adolescents. This provides a strong developmental rationale for utilizing the more concrete and age-appropriate BSCS-9.

The study results show that self-control positively predicts responsibility. However, the latent mediation model revealed that self-control accounts for only a small part of the variance in responsibility (8.6%). Given this modest effect size, this predictive relationship should be interpreted conservatively and not overinterpreted. This limited explanatory power calls for a deeper developmental evaluation. While self-control reflects the internal capacity to manage impulses and act on internalized values (Koç et al., 2023), responsibility is a complex concept inherently connected to social expectations and relevant guidelines that direct behavior (Schlenker et al., 1994). In early adolescence, internalizing responsibility is closely linked to moral development, as students shift from externally imposed rules to an autonomous stage (Akçinar et al., 2018; Piaget, 1932). Therefore, a teenager might have the cognitive self-control to resist an immediate urge, but internalizing responsibility heavily relies on cognitive growth (Damon and Gregory, 1997) and external support, such as family dynamics, cultural values, and peer influences (Finkenauer et al., 2005). This complex interaction explains why self-control is essential but not enough to fully predict personal responsibility, aligning with Mergler and Shield (2016), who viewed behavioral and cognitive control as only part of what determines adolescent responsibility.

Furthermore, the findings revealed that responsibility significantly mediates the relationship between self-control and patience. Patience is often mistakenly viewed as merely the absence of haste. However, the current findings support a more dynamic interpretation. Patience involves using self-control skills to handle stressors and daily interpersonal problems (Shubert et al., 2022). When adolescents develop a sense of personal responsibility, their capacity to delay gratification transforms from a simple act of self-denial into a value-driven endurance. Schnitker et al. (2017) demonstrated a strong positive link between adolescent patience and self-control, and our findings elaborate on this by positioning responsibility as the ethical bridge between them. This research confirms a positive connection among self-control, responsibility, and patience. In the context of educational sciences, practices focused on cultivating individual responsibility and self-control could positively influence adolescents’ character development (Cecchini et al., 2007). Educators and policymakers should consider that fostering self-control in isolation may not yield highly responsible individuals unless it is coupled with educational environments that promote moral autonomy and ethical reasoning.

### Limitations and directions for future research

Some limitations should be acknowledged in this research. First, four items (i.e., items 1, 9, 12, and 13) were omitted based on statistical analyses. This may be because younger age groups perceive the original scale, designed for adults, differently. Furthermore, a major limitation is relying on only one sample of 351 participants for both item refinement and validation. While using Rasch analysis with CFA for diagnostic purposes supports item removal, not having an independent cross-validation sample increases the risk of overfitting. Consequently, the current results should be interpreted as preliminary exploratory/confirmatory evidence rather than a definitive validation. Future research should replicate these findings with larger, more diverse samples to confirm the scale’s stability over time and its applicability to the Turkish population. Another limitation relates to the scale’s focus. The Rasch model revealed that the items better evaluate the lower end of the self-control spectrum than the higher end. This imbalance in what the scale measures suggests that the Turkish BSCS-9 should primarily be used as a screening tool for low self-control rather than as a measure of high self-control skills. Future studies should include more challenging items to enhance the scale’s ability to distinguish at higher levels of the trait.

Cultural and linguistic differences could also contribute to this outcome. Indeed, adaptation studies of the BSCS across various cultures have shown that the factor structures of the original scale vary due to cultural and linguistic nuances. Second, data on the psychometric properties of the BSCS were collected using self-report measures. Relying on self-reports may introduce social desirability bias or reflect temporary subjective opinions rather than stable behavioral traits. Although the data were collected from different secondary schools, the study used a cross-sectional design. Thus, the nine-item Turkish version of the BSCS could be validated longitudinally in different samples to assess its stability over time. Additionally, current reliability analyses of the Turkish BSCS-9 are limited to internal consistency coefficients. Future research should include test–retest reliability assessments to evaluate the scale’s short-term and long-term consistency. Finally, subsequent studies could explore the broader relationships involving adolescent self-control by examining its connections with contemporary variables, such as the psychological impacts of social media use or academic risk behaviors, offering a more comprehensive view of adolescent development in digital and educational settings.

## Conclusion

The Turkish version of the BSCS-9 is a unidimensional scale with strong psychometric properties, including confirmed measurement invariance across genders. It effectively assesses the self-control skills of Turkish adolescents aged 10 to 14 years. Beyond simply adapting a measurement tool, this study clarifies the complex interactions among self-control, responsibility, and patience during early adolescence. The findings indicate that self-control functions as a core cognitive ability that, when guided by personal responsibility, significantly improves an adolescent’s patience and capacity to handle daily challenges. Therefore, the BSCS-9 can be regarded as a valid and reliable instrument for future research. Educational professionals, school counselors, and researchers can use this scale to identify self-control needs, develop targeted interventions, and promote comprehensive character development in school settings.

## Data Availability

The raw data supporting the conclusions of this article will be made available by the authors, without undue reservation.
