# Supplementary material for: Psychometric properties, Rasch analysis, and measurement invariance of the Turkish Brief Self-Control Scale in early adolescents: exploring the mediating role of responsibility in the self-control and patience association
Source: Front Psychol. 2026 May 5;17:1829371. doi: 10.3389/fpsyg.2026.1829371 (PMC13183628; doi:10.3389/fpsyg.2026.1829371)
Supplement: Supplementary file 1 [file Data_Sheet_1.PDF]

Appendix A: The BSCS's Turkish Form

**Maddeler** (1: Bana Hiç Uygun Değil, 2: Bana Uygun Değil 3: Kararsızım, 4: Bana Uygun, 5: Bana Çok Uygun)	**Faktör Yükleri**
1. Nefsime karşı durma konusunda iyiyimdir. *	$.233^{***}$
2. Bana zarar veren alışkanlıklarımdan vazgeçmekte zorlanıyorum. (R)	$.486^{***}$
3. Üşengeç biriyim. (R)	$.462^{***}$
4. Çoğu zaman sonunu hesap etmeden konuşuyorum. (R)	$.502^{***}$
5. Eğer eğlenceliyse benim için kötü olan bazı şeyleri yaparım. (R)	$.667^{***}$
6. Keşke daha fazla kendime sahip olsaydım. (R)	$.395^{***}$
7. Eğlenceli durumlar bazen işimi yapmamı engelliyor. (R)	$.358^{***}$
8. Odaklanmakta (Konsantre olmakta) zorlanıyorum. (R)	$.506^{***}$
9. Kendime uzak hedefler koyarak etkili bir şekilde çalışabilirim. *	$.198^{**}$
10. Bazen yanlış olduğunu bilsem bile kendimi sevdiğim şeyler yapmaktan alıkoyamıyorum. (R)	$.513^{***}$
11. Genellikle düşünmeden hareket ederim. (R)	$.510^{***}$
12. Benim için kötü olan şeyleri reddederim. *	$.239^{***}$
13. Etrafımdakiler çelik gibi iradeye sahip olduğumu söyler. *	.123

Notlar. Tablodaki faktör yükleri, 13 maddelik ilk modelin WLSMV tahminleyicisi kullanılarak elde edilen tam standardize edilmiş (std.all) değerlerini göstermektedir. ***: $p < .001$, **: $p < .01$. R = Maddeler ters kodlanacaktır. * = Faktör yükleri 0.30'un altında olduğu için ölçekten çıkarılmıştır.

Appendix B: Category Probability Curves for Excluded Items

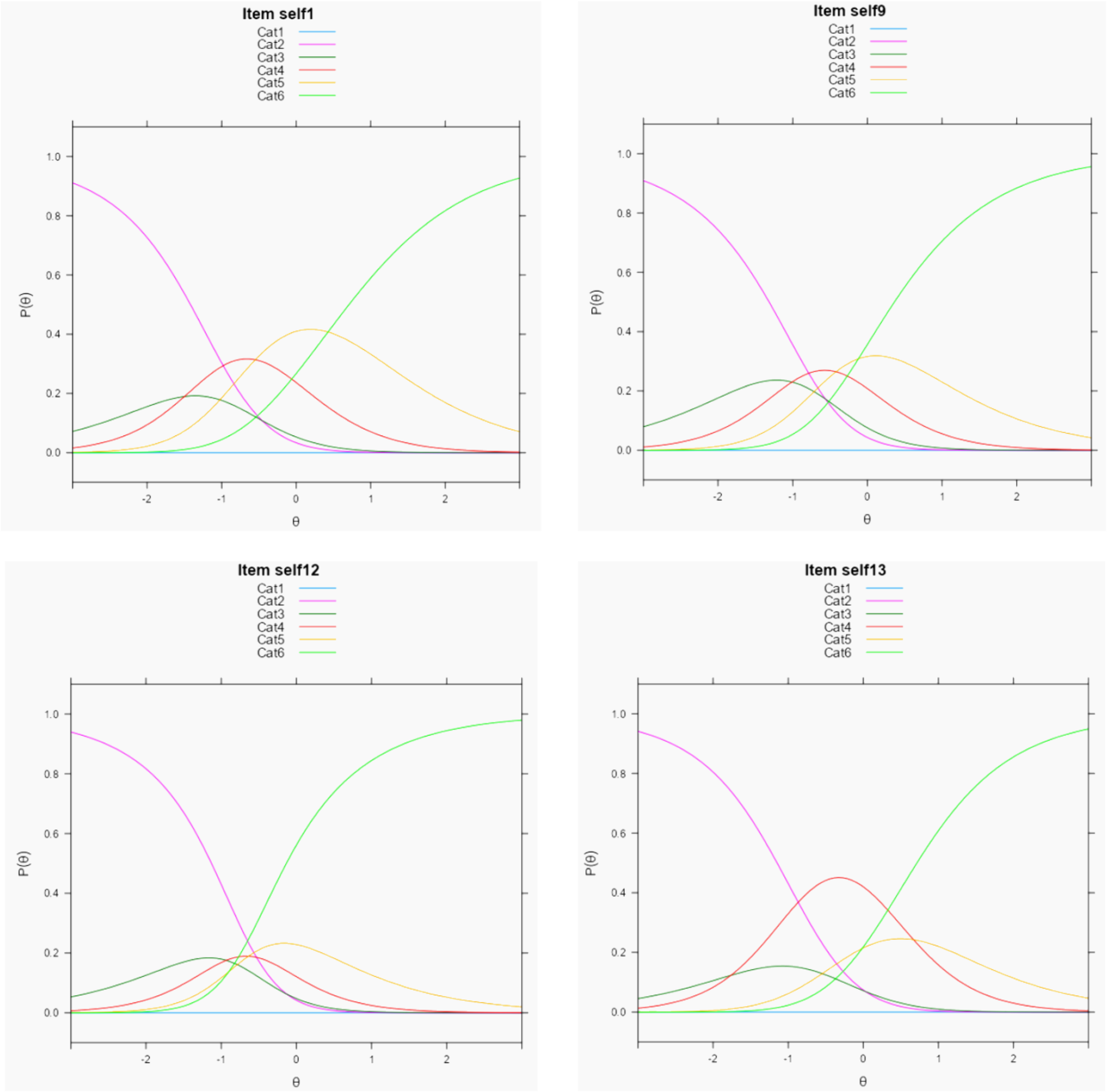

Note. The Category Probability Curves (CPC) were estimated based on the Polytomous Partial Credit Model (PCM) using Marginal Maximum Likelihood Estimation (MMLE) via the snowIRT module in Jamovi. The curves illustrate the probability of endorsing each response category across the latent trait (θ). As graphically evident, these underperforming items (Items 1, 9, 12, and 13) consistently exhibited disordered threshold (τ) parameters. Specifically, the estimated threshold parameters failed to increase monotonically across the rating scale (e.g., demonstrating reversals such as $\tau2 = 3.77$ to $\tau3 = 2.73$ for Item 1; $\tau2 = 3.57$ to $\tau3 = 3.13$ for Item 9; $\tau2 = 4.10$ to $\tau3 = 3.29$ for Item 12; and $\tau2 = 4.05$ to $\tau3 = 2.26$ for Item 13). Because multiple intermediate response categories failed to emerge as the most probable choice at any level of the latent trait, these statistical threshold reversals provide a clear, empirical justification for their exclusion from the final scale.

Appendix C: Polychoric Correlation Matrix for the BSCS-9 Items

Item	1	2	3	4	5	6	7	8	9
1. Item 2	—								
2. Item 3	.176	—							

3. Item 4	.270	.274	—						
4. Item 5	.373	.448	.342	—					
5. Item 6	.205	.237	.299	.248	—				
6. Item 7	.246	.220	.228	.237	.050	—			
7. Item 8	.365	.325	.312	.291	.275	.329	—		
8. Item 10	.304	.284	.219	.514	.207	.280	.219	—	
9. Item 11	.280	.203	.400	.404	.240	.200	.305	.420	—

Note. All values represent polychoric correlation coefficients estimated for the ordinal 5-point Likert items.
