# Supplementary material for: Psychometric properties, Rasch analysis, and measurement invariance of the Turkish Brief Self-Control Scale in early adolescents: exploring the mediating role of responsibility in the self-control and patience association
Source: Front Psychol. 2026 May 5;17:1829371. doi: 10.3389/fpsyg.2026.1829371 (PMC13183628; doi:10.3389/fpsyg.2026.1829371)
Supplement: Supplementary file 2 [file Table_1.docx]

**Appendix D:** *Results of the Principal Component Analysis (PCA) of Residuals*

| Contrast | Eigenvalue (λ) | Variance Explained (%) | Cumulative Variance (%) |
| --- | --- | --- | --- |
| 1 | 1.426 | 15.85 | 15.85 |
| 2 | 1.312 | 14.58 | 30.43 |
| 3 | 1.263 | 14.03 | 44.46 |
| 4 | 1.136 | 12.62 | 57.08 |
| 5 | 1.027 | 11.41 | 68.50 |
| 6 | 0.985 | 10.94 | 79.44 |
| 7 | 0.816 | 09.07 | 88.51 |
| 8 | 0.746 | 8.29 | 96.79 |
| 9 | 0.288 | 3.21 | 100.00 |

*Note.* First contrast eigenvalue (λ_1_) < 2.0 indicates that the unidimensionality assumption is met.

**Appendix E:** Differential Item Functioning (DIF) Analysis Results by Gender

| Item | Statistic (LR) | *p* | *padj​* |
| --- | --- | --- | --- |
| Item 2 | 0.859 | .651 | .837 |
| Item 3 | 6.261 | .044 | .131 |
| Item 4 | 0.134 | .935 | .935 |
| Item 5 | 10.855 | .004 | .040 |
| Item 6 | 0.277 | .871 | .935 |
| Item 7 | 1.733 | .420 | .748 |
| Item 8 | 4.964 | .084 | .188 |
| Item 10 | 6.675 | .036 | .131 |
| Item 11 | 1.393 | .498 | .748 |

*Note.* LR = likelihood ratio; *padj*​ = *p*-values adjusted for multiple comparisons.
